# Pure Drinking Water

**Published:** 1880-06

**Authors:** 


Pure Drinking Water.—Baltimore will soon be possessed of facilities for using river water to an unsurpassed extent, and probably when the great tunnel for supplying it from the Gunpowder river, is completed, and its capabilities rendered available to all classes of our citizens, it will not be equalled, even by any other city in the world. The supply will be as inexhaustible as bountiful, and facilities for bathing and cleansing purposes generally, must, on the completion of the extensive works, enter as conspicuous and important factors in the general hygiene of the city; but there is another factor that should not be lost sight of in estimating the intrinsic value of the water, viz: is it sufficiently free from mineral and other admixture, to warrant its continuous use for drinking and culinary purposes? Is it not possible, if not probable, that some, if not many, of the reported cases of heart disease, have had their origin in, or were increased, at least, by water which was supplied from Jones' Falls?

As these remarks are intended in some measure as introductory to what may be said on the character of our drinking water, by correspondents, and from this department also in future issues of the journal; we will close by reminding those of our readers who have promised their aid and co-operation in ventilating the subject in all its hygienic relations to this community, to let us have their favors as early as it may be convenient for them to do so.

